# L-alanine-induced germination in *Bacillus licheniformis* -the impact of native *gerA* sequences

**DOI:** 10.1186/1471-2180-14-101

**Published:** 2014-04-22

**Authors:** Elisabeth H Madslien, Per Einar Granum, Janet M Blatny, Toril Lindbäck

**Affiliations:** 1 Forsvarets Forskningsinstitutt FFI, Norwegian Defence Research Establishment, P. O. Box 25, N-2027 Kjeller, Norway; 2 Department of Food Safety and Infection Biology, Norwegian University of Life Sciences, P. O. Box 8146 Dep, N-0033 Oslo, Norway

**Keywords:** *Bacillus licheniformis*, Germination, L-alanine, *gerA*, Genotype, Germinant receptor

## Abstract

**Background:**

L-alanine, acting through the GerA receptor, was recently found to be an efficient germinant in *Bacillus licheniformis* ATCC14580/DSM13.

**Results:**

In this study, we show that several of 46 examined *B. licheniformis* strains germinate remarkably slower than the type strain when exposed to L-alanine. These strains are not necessarily closely related, as determined by MLST (multi-locus sequence typing). Three of the slow-germinating strains were further examined in order to see whether nucleotide substitutions in the *gerA* sequences were responsible for the slow L-alanine germination. This was performed by complementing the transformable type strain derivate MW3Δ*gerAA* with *gerA* variants from the three slow-germinating strains; NVH1032, NVH1112 and NVH800.

**Conclusions:**

A wide selection of *B. licheniformis* strains was evaluated for L-alanine-induced germination efficiency. Our results show that *gerA* substitutions could only partially explain why spores of some *B. licheniformis* strains responded slower than others in the presence of L-alanine.

## Background

Spores of *Bacillus licheniformis* and other *Bacillus* species are frequent contaminants in foods [1,2]. Exposure to nutrients triggers spores to leave dormancy in the process of germination [3-5]. This process involves several steps leading to rehydration of the spore core and loss of dormancy. Under favorable conditions, spores will grow out and multiply to numbers that can cause food spoilage and sometimes disease [6]. While dormant spores are extremely heat resistant, germinated spores can easily be killed by a mild heat treatment [7]. Therefore, a food preservation technique applied by food manufacturers to reduce spore numbers in food products is “induced germination”. The consequence of induced germination is spores germinated into vegetative cells will be heat sensitive and can therefore be inactivated, by successive heating below temperatures that compromise food quality (modified Tyndallization) [8-10]. The effectiveness of such a strategy depends on the germination rate of the spore population. A slow and/or heterogeneous germination rate of a specific spore population will reduce the effectiveness of such treatments [11-14].

Nutrient germinant receptors (GRs), localized to the inner spore membrane [15-17], are involved in the spore’s recognition of specific nutrients in its environment, which is the initial step in the spore’s return to growth [18]. Binding of nutrient to the receptors is believed to trigger the release of the spore core’s large depot of Ca-dipicolinic acid (CaDPA), followed by rehydration of the spore core and degradation of the spore cortex [3]. Current knowledge about GRs and their nutrient specificity is mainly achieved from *Bacillus subtilis* and *Bacillus cereus.* However, genes encoding GRs are widely distributed among *Bacillus* and *Clostridium* species [5,19], implicating an essential role in triggering of spore germination in most spore-forming bacteria. Interestingly, the nutrient specificity of the receptors and the interaction between them varies between and even within species, as has been shown for *B. cereus*-group members [20-22].

GRs are generally encoded by polycistronic operons that are expressed late in sporulation under the regulation of the forespore-specific transcription factor, sigma G (σ^G^) [23,24]. These genes constitute a family (*gerA* family) of homologous genes that probably have evolved from the same ancestor [4,19]. Three putative *gerA* family operons, *gerA* (*A, B, C*)*, gerK* (*A, C, B*) and *ynd* (*D,E*_*3*_*E*_*2*_*, F*_*1,*_*E*_*1*_) and the single *gerAC* homologue *yndF2* have been identified within the *B. licheniformis* type strain ATCC14580/DSM13 genome [25-27]. Of these, only the *gerA* operon has been functionally characterized so far [28]. *gerA* was found to be essential for germination in presence of L-alanine. A similar role has been described for *gerA* in *B. subtilis*[18]*.* L-alanine is probably the most universal single nutrient germinant among spore formers [19].

The *Bacillus* GRs which have been described so far are usually composed of three subunits termed A, B and C. The A and B subunits are predicted to contain 5–6 (A) and 10–11 (B) membrane-spanning domains, respectively [5,29], while the C subunit is thought to be a membrane-anchored lipoprotein [30]. The tertiary structure of *B. subtilis* GerBC was determined a few years ago [31]. The B-subunit, whose amino acid sequence shows homology to proteins of the APC (amino acid-polyamine-organocation) superfamily, is proposed to be the most likely site of ligand binding, as mutations within this subunit alter ligand specificity [4,32]. However, since mutations in any of the three cistrons are shown to disturb receptor function, the exact site of nutrient binding is still unknown [5].

The genetic relationship of 53 strains of the food-spoilage agent *B. licheniformis,* a close relative of *B. subtilis,* was recently described by a novel MLST scheme [33]. One of these strains, NVH1032, was isolated after surviving an “induced germination”-regime (Tyndallization), applied by the food industry to eliminate spore contamination. Preliminary results in our lab suggested that NVH1032 and other *B. licheniformis* strains germinate considerably slower than the type strain when exposed to L-alanine. Such slow-germinating strains pose a challenge to food manufacturers that want to implement “induced germination” as a strategy to reduce/eliminate spores during processing.

In this study, 46 of the 53 genotyped strains were screened for efficiency of L-alanine-induced germination, and the correlation between the genotype and the induced germination was determined. Furthermore, it was investigated whether the slow germination of three particular *B. licheniformis* strains was due to sequence differences in the *gerA* operon.

## Results and discussion

### Screening of L-alanine-induced germination in *B. licheniformis* strains

In order to evaluate the efficiency of L-alanine-induced germination of the 46 *B. licheniformis* strains, the level of germination was recorded after addition of L-alanine in a screening assay. The results showed that germination efficiency, determined by reduction of absorbance (A_600_) varied from ~1 to 60% between the tested strains 2 h after the addition of germinant (Additional file 1). A drop in A_600_ of 60% was equivalent to > 95% germinated spores, as verified by phase contrast microscopy. About 30 of the strains germinated well with a reduction in absorbance of 40% or more, while six strains germinated poorly (10% or less in reduction of absorbance).

In general, differences in germination between strains may be due to differences in lag time (interval between addition of germinant and loss of refractivity) and differences in rate of germination (slope of the germination curve/∆A_600_ min^-1^). Several factors may account for these differences: (i) permeability of the outer spore layers, restricting access of germinant to the inner membrane [34], (ii) germinant specificity [20,22], (iii) GR (nutrient germinant receptors) level [35], (iv) dysfunctional GRs [36], (v) GR synergism/antagonism [37] and/or (vi) structure of the cortex [38]. Within single populations of *B. subtilis,* a reduced level of GRs has been suggested to be one of the main reasons for slow germination or “superdormancy” [35], probably by increasing the lag time until CaDPA is released [14]. In *B. subtilis,* GRs have been proposed to be present in a relatively low number (<40) in the spore’s inner membrane where they form discrete clusters, so-called germinosomes [16,39], however, it has recently been reported that this number may be highly underestimated [40]. The number of germination receptors has been shown to be strongly dependent on the sporulation conditions [4,41,42]. In this study, sporulation and germination conditions (e.g. temperature, sporulation medium, pH, activation time/temperature, germinant concentration) were optimized with respect to the type strain ATCC14580/DSM13. However, these conditions may not be optimal for all strains.

### Distribution and characterization of the *gerA* operon

**Figure 1:**
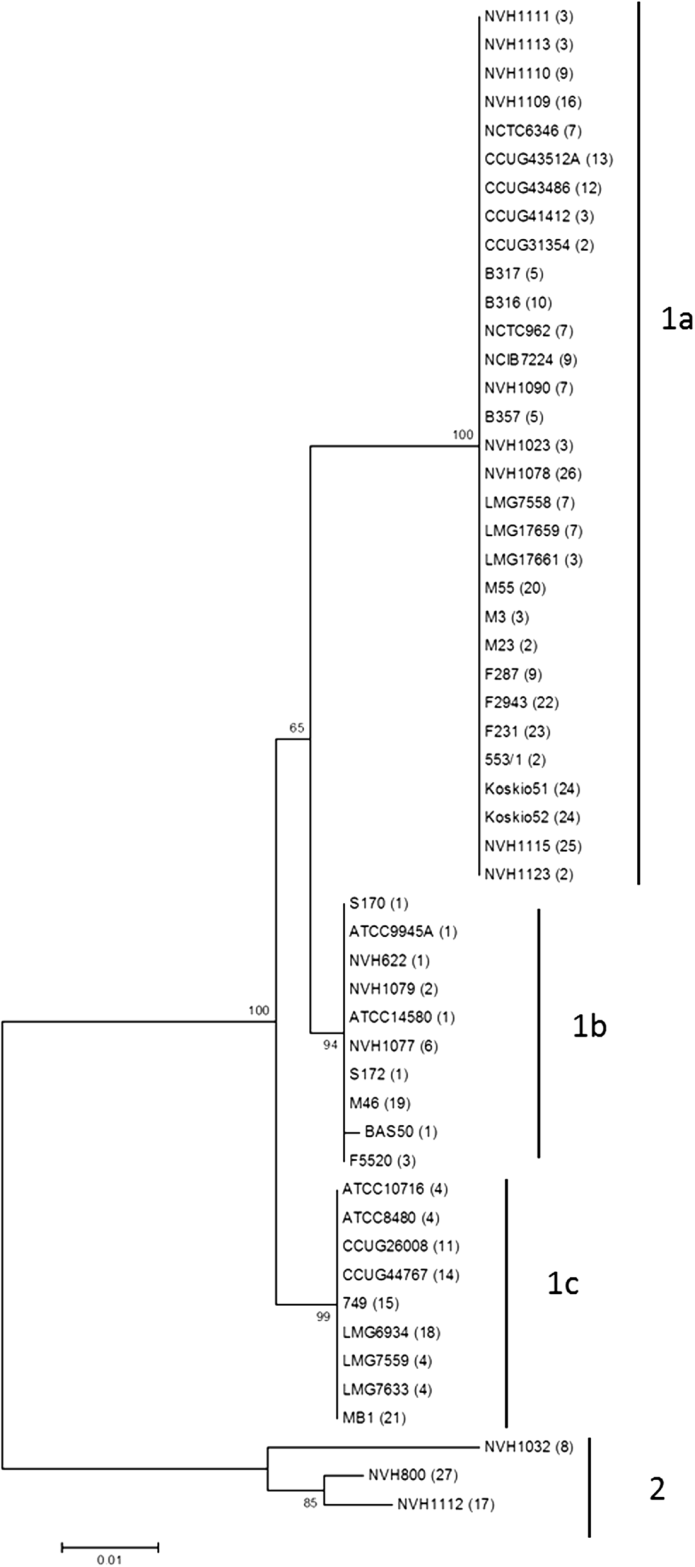
**Cluster analysis of partial *gerA *sequences from 53 *B. licheniformis *strains.** Dendogram of partial *gerA* operon sequences (626 bp) in 53 *B. licheniformis* strains. The sequences cover parts of the last two genes (*gerAB* and *gerAC*) of the tricistronic *gerA* operon. The dendogram was calculated using the NJ- method with tree branch quality assessed using bootstrap values (500 replicates) as shown next to the branches. The evolutionary distances were computed using the Maximum Composite Likelihood method and are in the units of the number of base substitutions per site. MLST sequence type (ST) is indicated in brackets behind each strain and *gerA* cluster (1a, b, c and 2) is indicated with solid vertical lines to the right. Analyses were conducted in MEGA5.

The *gerA* locus was detected by PCR in all of the 53 genotyped *B. licheniformis* strains (GenBank: KF358523- KF358575). To investigate whether certain *gerA* sequence variants were associated with slow germination, partial *gerA* operon sequences of all strains were analysed, aligned and organized into clusters. The resulting neighbour-joining (NJ) tree is presented in Figure 1. With the exception of two strains (NVH1109/“1a” and NVH1077/“1b”) the NJ- dendogram was congruent with the MLST tree generated from six house-keeping genes [33]. Thus, the *gerA* locus seemed to have evolved in parallel to the house-keeping genes. The ratio of non-synonymous versus synonymous base substitutions (d*N*/d*S)* was 0.0845 which is somewhat higher than the calculated values for the individual MLST loci (0.0000-0.0457) [33], but far below the limit of 1.0 that is often set for loci undergoing positive selection. Thus, the *gerA* locus, similar to the house-keeping genes, seems to be subject to purifying (stabilizing) selection [43,44].

A total of seven unique alleles were distributed into four main clusters, determined “1a”, “1b”, “1c” and “2” (Figure 1). Cluster “2” was represented by only three strains, NVH1032, NVH800 and NVH1112, that all showed a slower and less efficient germination response (Additional file 1) compared to the type strain, ATCC14580/DSM13 (cluster “1b”). However, slow-germinating strains were also found within each of the other clusters. Thus, this part of the *gerA* operon sequence (718 bp ranging from 3′ end of *gerAB* to 5′ end of *gerAC*) was not suitable in order to completely distinguish slow-germinating and fast-germinating strains.

### Germination of *gerA* complementation strains

**Figure 2:**
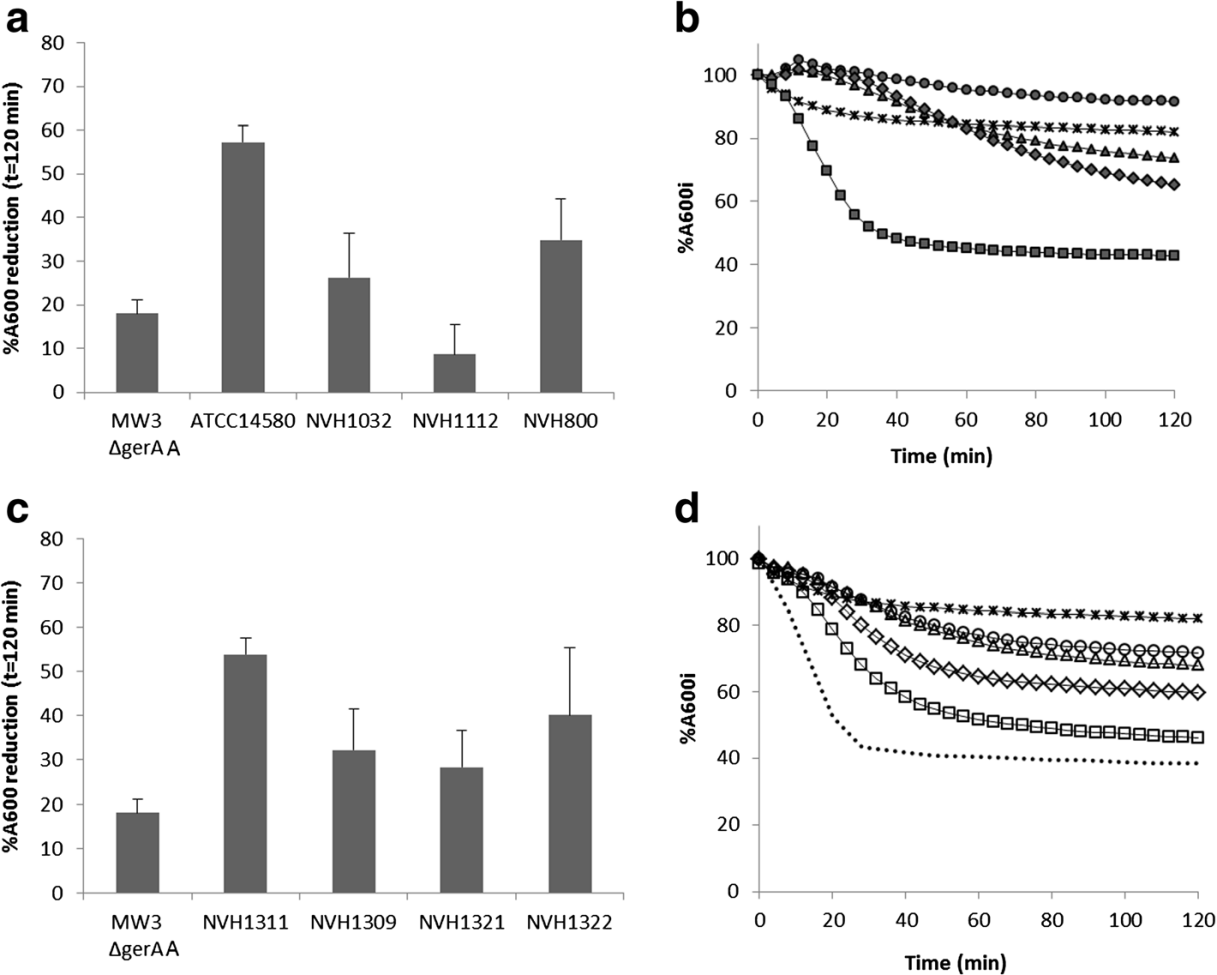
**Spore germination of slow-germinating strains and of *gerAA *disruption mutant complemented with *gerA *sequences from slow-germinating strains. ab**: Germination of MW3∆*gerAA* (x), the wild-type strains ATCC14580 (■), NVH 1032 (▲), NVH1112 (●) and NVH800 (♦) measured as reduction in absorbance (A_600_) after addition of germinant (100 mM L-alanine). **cd**: Spore germination of the MW3∆*gerAA* (x), and MW3∆*gerAA* complemented with *gerA* from ATCC14580 (□ NVH1311), NVH1032 (∆ NVH1309), NVH1112 (○ NVH1321) and NVH800 (◊ NVH1322) measured as reduction in absorbance (A_600_) after addition of germinant (100 mM L-alanine). The results represent the average (SD) of three independent spore batches. The type strain derivate MW3 (dotted line) has been included in Figure 3D for comparison.

In order to further investigate the influence of *gerA* sequences on germination rate, MW3Δ*gerAA* was complemented with *gerA* operons originating from the type strain ATCC14580/DSM13 [28], and the three slow-germinating strains (Figure 2c,d). The *gerA* sequences of ATCC14580/DSM13 , NVH1032 and NVH800 nearly restored the phenotype of the sequence originating strains, while complementing MW3∆*gerAA* with the *gerA* sequence from NVH112 increased the germination rate of the complemented strain compared to NVH1112 wild-type (Figure 2a,c). Still, the order of the germination rate between the four strains was consistent between the two experiments (NVH1112/NVH1321 < NVH1032/NVH1309 < NVH800/NVH1322 < ATCC14580/NVH1311), substantiating that the phenotypes of the complemented MW3∆*gerAA* mutant to some extent restored the phenotypes of the *gerA* originating strains. Germination data of MW3 carrying pHT315 (MW3_pHT315) showed that carrying the empty vector, or the use of erythromycin in the cultures, hampered the germination rate of the MW3 strain (Additional file 2). However, we assume that comparing the effect of the complementing sequences is acceptable since they are all carried by the same vector.

An important observation was that, in contrast to Løvdal *et al.* 2012 [28], L-alanine-induced germination was not completely abolished in MW3∆*gerAA* (NVH1307). This weak germination (~10% phase dark spores after 120 min) was not observed in absence of germinant, indicating that germination receptors other than GerA might be weakly activated by L-alanine. We also noted that spores of the slow-germinating strain NVH1112 hardly germinated at all, and to a lesser extent than MW3∆*gerAA* (Figure 2a,b). When complementing MW3∆*gerAA* with the *gerA* operon from NVH1112 (NVH1321) germination efficiency increased, indicating that the *gerA* operon of NVH1112 has some functionality in presence of L-alanine. A faster and more efficient germination of the complementation mutants compared to their respectively *gerA* originating strains was also observed for NVH1322 (*gerA* from NVH800) and NVH 1309 (*gerA* from NVH1032).

**Figure 3:**
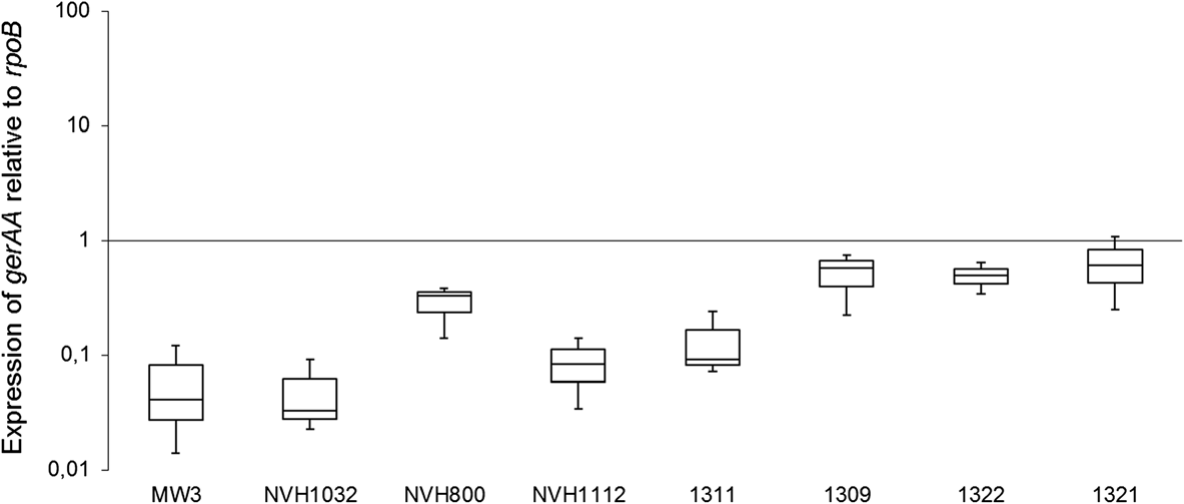
**Relative gene expression of *gerAA.*** Transcription level of *gerAA* relative to *rpoB* determined by qRT-PCR in *B. licheniformis* MW3, *B. licheniformis* NVH1032, *B. licheniformis* NVH 800, *B. licheniformis* NVH1112, and MW3∆*gerAA* complemented with *gerA* from the four abovementioned strains. The horizontal line in the box represents the median expression value, and the box encompasses 50% of the observations (first quartile (Q1) to third quartile (Q3)). The ends of the whisker are set at 1.5*IQR above the third quartile and 1.5*IQR below the first quartile.

The imperfect complementation of the phenotypes may be due to several different factors. Firstly, a two- to seventeen-fold increase in expression level of *gerAA* was observed when MW3∆*gerAA* was complemented with different *gerA* sequences and compared to the wild-type strains from where the *gerA* sequences originated (Figure 3). The increased *gerAA* expression level in the complementation mutants might be related to the copy-number of the plasmid pHT315 (15 copies per cell). Previous experiments have shown that a 2–200 fold overexpression of *ger* genes may increase germination rate [45,46].

Secondly, since the complementing *gerA* genes in this experiment were plasmid-born (pHT315 encoding erythromycin resistance), 1 μg ml^-1^ erythromycin was used in the sporulation medium to maintain the plasmid throughout the sporulation process and MW3 carrying the pHT315 empty vector germinated slower and with less efficiency than the wild-type MW3 strain (Additional file 2). Despite this observation, MW3∆*gerAA* complemented with slow germinating *gerA* sequences germinated better than the strains from where the *gerA* sequences originated (Figure 2a-d).

Thirdly, the entire *gerA* operon and the 151 bp region upstream of the start codon of *gerAA* was cloned in the complementing vector pHT315. Alignments of the promoter sequence of strain NVH1032, NVH800, NVH1112 and ATCC14580/MW3 can be viewed in Additional file 3. No differences were observed between the type-strain and the slow germinating strains in the -10 and -35 promoter region of *gerA*. However, differences outside these regions may influence the transcriptional level. pHT315 [47] contains the inducible *lac* promoter, but transcription from this promoter cannot be excluded even without induction.

Despite the imperfect restoration of the wt phenotypes, the results of the germination assays in this study indicate that the *gerA* sequences have an impact on germination rate and efficiency. Differences in the GerA amino acid sequence may lead to altered protein 3-D structure, which again may cause impaired assembly and stability of the receptor complex in the inner membrane, lower or higher substrate affinity or influence the interactions with other membrane proteins.

### Structural analysis of amino acid substitutions in the GerA receptor

Analyses of single amino acid substitutions have previously been conducted in *B. subtilis* GerAA [48], GerAB [49] and GerBC [50]. None of these positions were substituted in the GerA sequences examined in the present study. Alignments of the GerAA, GerAB and GerAC sequences of *B. licheniformis* NVH1032, NVH800, NVH1112 and ATCC14580/DSM13 are presented in Additional files 4, 5 and 6. Thus, on the basis of this knowledge and the lack of a 3D structure of any proteins in the GerAA and GerAB families of proteins, the relevance of the observed differences within these two subunits is difficult to determine. However, the crystal structure of *B. subtilis* GerBC has recently been determined [31]. Using this structure as a template for prediction of *B. licheniformis* GerAC structures, one of the perhaps most interesting substitutions is F342S (NVH1032 and NVH800) which lies in the so-called “region 2” of domain III [50] (Additional file 7). Region 2 is reported to be a well conserved region in GerBC among *Bacillales* and substitutions within this region were previously shown to affect receptor function in *B. subtilis*[50]*.* On the other hand, the F342S substitution was neither observed in the *gerA* sequences of the slowest germinating strain NVH1112 or the fastest germinating strain ATCC14580/DSM13 suggesting that the role of this site seems unclear. It should be mentioned that the aa sequence of the GerAC protein of NVH1112 is much closer to that of MW3 than the other two (Additional file 6), indicating that GerAC is not crucial to germination efficiency. Ultimately, the lack of information about the exact germinant binding site, as well as the fact that only the C subunit has been structurally characterized, makes it difficult to interpret the effect of single substitutions on the GerA receptor function.

## Conclusions

This study shows that spores of 46 *B. licheniformis* strains are able to germinate in the presence of L-alanine, but that the germination rate and efficiency differ significantly between the strains. About 10% of the strains germinated poorly, even in presence of high (100 mM) concentrations of probably the most universal and potent germinant for *Bacillus* species in general, and *B. licheniformis* in particular. Germination rate of different bacterial strains are of importance to the food industry, using so-called “induced germination”, eg Tyndallization, to decrease spore contamination in processed foods. Delayed germination may reduce the efficiency of Tyndallization by allowing ungerminated spores to survive. Our results demonstrate that nutrient-induced germination followed by inactivation can be challenging when dealing with specific *B. licheniformis* strains*.*

The germination phenotype was partly restored when complementing a *gerAA* disruption mutant with *gerA* operons from either slow- or fast-germinating *B. licheniformis* strains. This observation indicates that differences in *gerA* family operons are partly responsible for differences in germination efficiency of *B. licheniformis* in response to L-alanine.

## Methods

### Strains

Strains included in this work are listed in Table 1. The 53 strains were previously characterized and genotyped by a novel MLST scheme [33].

**Table 1:** Strains used in this study

**Strain**	**Description**	**Reference**
**MW3**	*B. licheniformis* DSM13 (Δ*hsdR1,ΔhsdR2*)	[51]
**NVH1307**	*B. licheniformis* MW3Δ*gerAA::spc. SpR*	[28]
**NVH1311**	NVH1307 with pHT315_MW3*gerA. SpR* and *ErmR*	[28]
**NVH1309**	NVH1307 with pHT315*_*NVH1032*gerA. SpR* and *ErmR*	This work
**NVH1321**	NVH1307 with pHT315*_*NVH1112*gerA. SpR* and *ErmR*	This work
**NVH1322**	NVH1307 with pHT315*_*NVH800*gerA. SpR* and *ErmR*	This work
**53 *B. licheniformis *** **strains**	Genotyped wt strains from various sources	[33]

MW3 ∆*gerAA* (NVH1307) and the complementation mutant NVH1311 are described in Løvdal *et al.* 2012 [28]. The complementation mutants NVH1309, NVH1321 and NVH1322 were constructed in this work as described later on.

### DNA extraction

Bacteria were grown on sheep blood agar at 30°C overnight. Single colony material was inoculated in 20 mL Luria broth (LB). The bacterial culture was grown overnight at 30°C and centrifuged at 3000 × g for 10 min. The supernatant was discarded and the pellet resuspended in 1 mL enzymatic lysis buffer (20 mM Tris · Cl, pH 8.0, 20 mM Tris · Cl, pH 8.0, 1.2% Triton® X-100, 20 mg mL^-1^ lysozyme (Sigma, Steinheim, Germany)). Further DNA extraction was performed according to the protocol provided by DNeasy Blood and Tissue Kit (Qiagen, USA).

### PCR and sequencing of the *gerA* operon

Primer A7F and A7R (Table 2) were used to amplify a 718 bp region of the *gerA* operon, including 3′ end of *gerAB* and 5′ end of *gerAC*. Additionally, complete *gerA* operons from strain NVH800, NVH1032 and NVH1112 were amplified in smaller fragments for DNA sequencing using primers listed in Additional file 8. All amplification reactions were performed in 20 μL using 2 μL DNA (10 ng μL^-1^) as a template. PCR reactions were performed in a LightCycler® 480 System using LightCycler® 480 SYBR Green I Master (Roche Diagnostics GmbH, Germany) according to recommendations given by the manufacturer of the kit. The temperature program was as follows: 5 min initial denaturation at 95°C followed by 35 cycles of denaturation at 95°C for 10 s, annealing at 56°C for 10 s and extension at 72°C for 30 s. The amplifications were terminated after a final elongation step of 7 min at 72°C. The PCR fragments were verified by electrophoresis using Bioanalyzer (Agilent Technologies, USA). PCR products were purified and sequenced by Eurofins MWG Operon (Ebersberg, Germany) using the dideoxy chain termination method on an ABI 3730XL sequencing instrument (Applied Biosystems, USA).

**Table 2:** Primers used in this study

**Primer**	**Sequence**	**Application**	**Amplicon size**
**A7F**	5′- GGATTTGGGATACCGCTCTT -3′	*gerA* detection/sequencing	718 bp
**A7R**	5′- TGCAGATGCTGCGAGAATAC -3′	*gerA* detection/sequencing	718 bp
**gerAAF MW3**	5′- CCCTGTTCCTATCGGCGTTT -3′	RT-PCR (E = 2.01)	59 bp
**gerAAR MW3**	5′- TCGGCAGCATGCCTTGA -3′	RT-PCR (E = 2.01)	59 bp
**gerAAF 1112/1032/800**	5′- CGCCGTTCCCACAGATTC –3′	RT-PCR (E = 2.01/1.98/1.95)	55 bp
**gerAAR 1112/1032/800**	5′- CAGCGCTGAAGAAACCTTGTC –3′	RT-PCR (E = 2.01/1.98/1.95)	55 bp
**rpoBF**	5′- ACCTCTTCTTATCAGTGGTTTCTTGAT -3′	RT-PCR (E = 2.00)	70 bp
**rpoBR**	5′- CCTCAATTGGCGATATGTCTTG -3′	RT-PCR (E = 2.00)	70 bp

### Data analysis

The Staden Package [52] was used for alignment, editing and construction of consensus sequences based on the ABI sequence chromatograms. Consensus sequences (626 bp) were entered into the MEGA5 software [53] and aligned by CLUSTALW [54]. Dendograms were constructed in MEGA5 using the Neighbor-Joining method (NJ) [55] with branch lengths estimated by the Maximum Composite Likelihood method [56]. Branch quality was assessed by the bootstrap test using 500 replicates. Sequences were trimmed to be in frame, which means that eight bases in the transition between *gerAB* and *gerAC* were removed, before entering into S.T.A.R.T. 2 [57]. This program was used to calculate the d*N/*d*S* ratio (ratio of nonsynomous versus synonymous substitutions) [58].

The *B. licheniformis gerA* promoter sequence was identified in DBTBS [59] and prediction of transmembrane α-helices of GerAA and AB was performed using TOPCONS web program [60]. Finally, three-dimensional (3D) structure modeling of GerAC was performed using RaptorX and PyMOL [61,62]. All sequences were compared against the annotated sequence of the *gerA* operon (*gerAA, gerAB, gerAC*) of *B. licheniformis* ATCC14580/DSM 13 (YP_080584.1; YP_080585.1; YP_080586.1) [25] and *B. subtilis* subsp. *subtilis* str. 168 (NP_391185.2; NP_391186.1; NP_391187.1) [23,63].

### Construction of *B. licheniformis* MW3∆*gerA* complementation mutants

The entire *gerA* operons including the putative *sigG* promoter from *B. licheniformis* strain NVH1032, NVH800 and NVH1112 were cloned into the pHT315 [47] shuttle vector and introduced into the *gerAA* deletion mutant strain MW3∆*gerAA* by electroporation as described previously [28]. Briefly, PCR, with primers (Table 2) containing *Sal*I and *Xba*I restriction sites, was used to amplify the *gerA* operon including 151 bp upstream of the *gerAA* start codon and 177 bp downstream of the *gerAC* STOP codon. The amplified fragments were cloned into the *Sal*I/*Xba*I restriction site of pHT315, giving the complementation plasmids. For details regarding primers, PCR conditions, DNA isolation and electroporation see Løvdal *et al.* 2012 [28]. The strains created in this study were designated as follows: *B. licheniformis* NVH1309 (MW3∆*gerAA* _NVH1032*gerA*); NVH1321 (MW3∆*gerAA* _NVH1112*gerA*) and NVH1322 (MW3∆*gerAA* _NVH800*gerA*). Correct construction of the complementation plasmids was confirmed by sequencing and the complementation mutants were verified by PCR analysis. Sequence editing and alignments were performed as already described in the Data analysis section.

### Bacterial growth and sporulation

Sporulation was performed according to Løvdal *et al.* 2012 [28], with minor modifications. Bacteria were pre-cultured overnight in LB-broth with agitation (230 rpm) at 37°C. Complementation mutants were grown in presence of 1 μg mL^-1^ erythromycin. 10 μL of preculture was transferred to 50 mL of the non-defined, rich sporulation medium [28] in 500 mL EM flasks. Incubation was performed with agitation (230 rpm) at 37°C for 3–7 days until ≥ 80% phase bright spores as judged by phase contrast microscopy. Seven of the strains (M55, ATCC9945A, NVH622, 749, M46, NVH1079 and LMG6934) did not sporulate adequately and were excluded from further analysis. Spores were harvested by centrifugation for 10 min at 3900 × *g* (Eppendorf) at 4°C and resuspended in 10 mL ice-cold autoclaved Milli-Q water. The spores were centrifuged at 10000 × *g* through a 50% (w/v*)* Nycodenz (Axis-Shield) gradient in order to remove cell debris and vegetative cells. The spores were washed three times in ice-cold autoclaved Milli-Q water before storage (1–3 months) in the dark at 4°C. The final spore suspensions were 98% free of vegetative cells, not fully sporulated cells, cell debris and germinated cells as judged by phase contrast microscopy.

### Quantitative RT-PCR

Quantitative RT-PCR experiments were performed on mRNA isolated from *B. licheniformis* cultures harvested after ~ 50% sporulation judged by phase contrast microscopy. Total bacterial RNA was extracted using TRIzol Reagent (Invitrogen) and cells were disrupted using Lysing Matrix B (MP Biomedicals Europe) and bead beating in a Mini-BeadBeater-8 (BioSpec) according to manufacturer’s specifications. DNA was removed from each RNA preparation using Turbo DNA-free Kit (Ambion), according to manufacturer’s instructions. RNA quantity (A260) and purity (A260/280 ratio) were measured in a NanoDrop 1000 Spectrophotometer (Thermo Fisher Scientific). cDNA was synthesised from 500 ng RNA using the High-Capacity cDNA Reverse Transcription Kit (Applied Biosystems) in a 20 μl reaction according to manufacturer’s protocols.

Five μl of a 1:100 dilution of the cDNA reaction was used as template for qPCR amplification in 25 μl final volumes containing 12.5 μl of Power SYBR Green PCR Master Mix (Applied Biosystems) and 200 nM of each primer. Primers used for qPCR are listed in Table 2. The amplification was performed using StepOne PCR software (Applied Biosystems) with thermal cycling conditions set at 10 min at 95°C, followed by 40 cycles of 15 s at 95°C and 1 min at 60°C. Fluorescence was monitored during each extension phase and a melting curve analysis was performed after each run to confirm the amplification of specific transcripts. Each qPCR of the RNA samples was performed in triplicate, no template was added in negative controls, and *rpoB* was used as internal control. The qPCR analysis was performed on three independent biological replicates. Slopes of the standard curves and PCR efficiency (E) for each primer pair were estimated by amplifying serial dilutions of the cDNA template. For quantification of mRNA transcript levels, Ct (threshold cycle) values of the target genes (*gerAA*) and the internal control gene (*rpoB*) derived from the same sample in each real-time PCR reaction were first transformed using the term E^-Ct^. The expression levels of target genes were then normalized by dividing their transformed Ct-values by the corresponding values obtained for internal control gene [64,65].

### Germination assays

Storage water was decanted and the spores were resuspended in autoclaved Milli-Q water (20°C) immediately before heat activation at 65°C in a heating block (QBD2, Grant Instruments Ltd) for 20 min. The heat-activated spores were rapidly cooled down by centrifugation for 3 min 4500 × *g* at 4°C before resuspension in germination buffer (200 mM K-phosphate buffer pH 7.2). The A_600_ of the buffered spore suspension was adjusted to ~2.1 (Shimadzu UV- 160A, Shimdazu Europe GMBH). L-Alanine (Sigma) was dissolved in Milli-Q water and filter sterilized prior to use through a 0.45 μm pore size filter. 100 μL of 0.05 - 0.2 M L-Alanine germinant solution was added to 100 μL buffered spore suspension in a 96-well microplate (BD) giving an initial A_600_ of ~1. Germination was by monitored by reading the drop in absorbance (A_600_) in a 96-well microplate reader (Tecan Infinite M200). Readings were performed at regular intervals (2 min) and the plate was shaken 10 s prior to each reading. Set point temperature during germination was 37°C (36.5 - 37.5). The screening of 46 strains was performed in duplicate with a single spore preparation. All other experiments were performed with three independent spore preparations.

## Competing interests

The authors declare that they have no competing interests.

## Authors’ contributions

All authors contributed to the design of the study. EHM drafted the manuscript, assisted in the construction of the complementation mutants and performed the germination experiments, PCR amplifications, sequence editing, sequence alignments and data analysis. JMB and PEG assisted in drafting the manuscript. TL performed the RT-PCR experiments, constructed the complementation mutants and assisted in data analysis and drafting the manuscript. All authors have read and approved the final version of the manuscript.

## Supplementary Material

Additional file 1 **Comparison of germination efficiency in 46 *B. licheniformis *strains.** The relative decrease in absorbance (A_600_) in the spore suspension was measured 2 h after the addition of germinant (100 mM L-alanine). The strains NVH1032, NVH800, ATCC14580/DSM13 and NVH1112 were selected for further analysis (indicated with arrows). Click here for file

Additional file 2 **Spore germination of MW3 carrying pHT315.** Germination of MW3 (▲) and MW3_pHT315 () measured as reduction in absorbance (A_600_) after addition of germinant (100 mM L-alanine). MW3_pHT315 ctrl (■) is not added any germinant. Click here for file

Additional file 3 **Promoter sequence alignment.** Alignment of the estimated σ^G^ dependent *gerA* promoter sequences of *B. subtilis* spp. *subtilis* str.168 and *B. licheniformis* ATCC14580/DSM13, NVH1112, NVH800 and NVH1032. DBTBS was used to identify promoter sequences. The *B. subtilis* promoter (underlined) and transcriptional start site (arrow) were experimentally defined by Feavers *et al*. (1990) [24]. Click here for file

Additional file 4 **Amino acid sequence alignment of GerAA from ATCC14580/DSM13, NVH1032, NVH800 and NVH1112.** Residues with substitutions are indicated in yellow. Alignment was performed with ClustalW in MEGA5. The numbered solid lines indicate regions of predicted transmembrane domains (TOPCONS). Click here for file

Additional file 5 **Amino acid sequence alignment of GerAB from ATCC14580/DSM13, NVH1032, NVH800 and NVH1112.** Residues with substitutions are indicated in yellow. Alignment was performed with ClustalW in MEGA5. The numbered solid lines indicate regions of predicted transmembrane domains (TOPCONS). Click here for file

Additional file 6 **Amino acid sequence alignment of GerAC from ATCC14580/DSM13, NVH1032, NVH800 and NVH1112.** Residues with substitutions are indicated in yellow. Alignment was performed with ClustalW in MEGA5. Click here for file

Additional file 7 **3D-model of the GerAC protein of *B. licheniformis.*** Substitutions that were detected in strain NVH1032, NVH800 and NVH1112 are indicated with red. Modelling was performed in PyMOL. Click here for file

Additional file 8 **Primers used in PCR amplification and DNA sequencing of *gerA *** **operons from *B. licheniformis *** **strains NVH 1112, NVH1032 and NVH800.** Click here for file
