# Supplementary figures and images for: L-alanine-induced germination in Bacillus licheniformis -the impact of native gerA sequences
Source: BMC Microbiol. 2014 Apr 22;14:101. doi: 10.1186/1471-2180-14-101 (PMC4021175; doi:10.1186/1471-2180-14-101)

## Slide 1
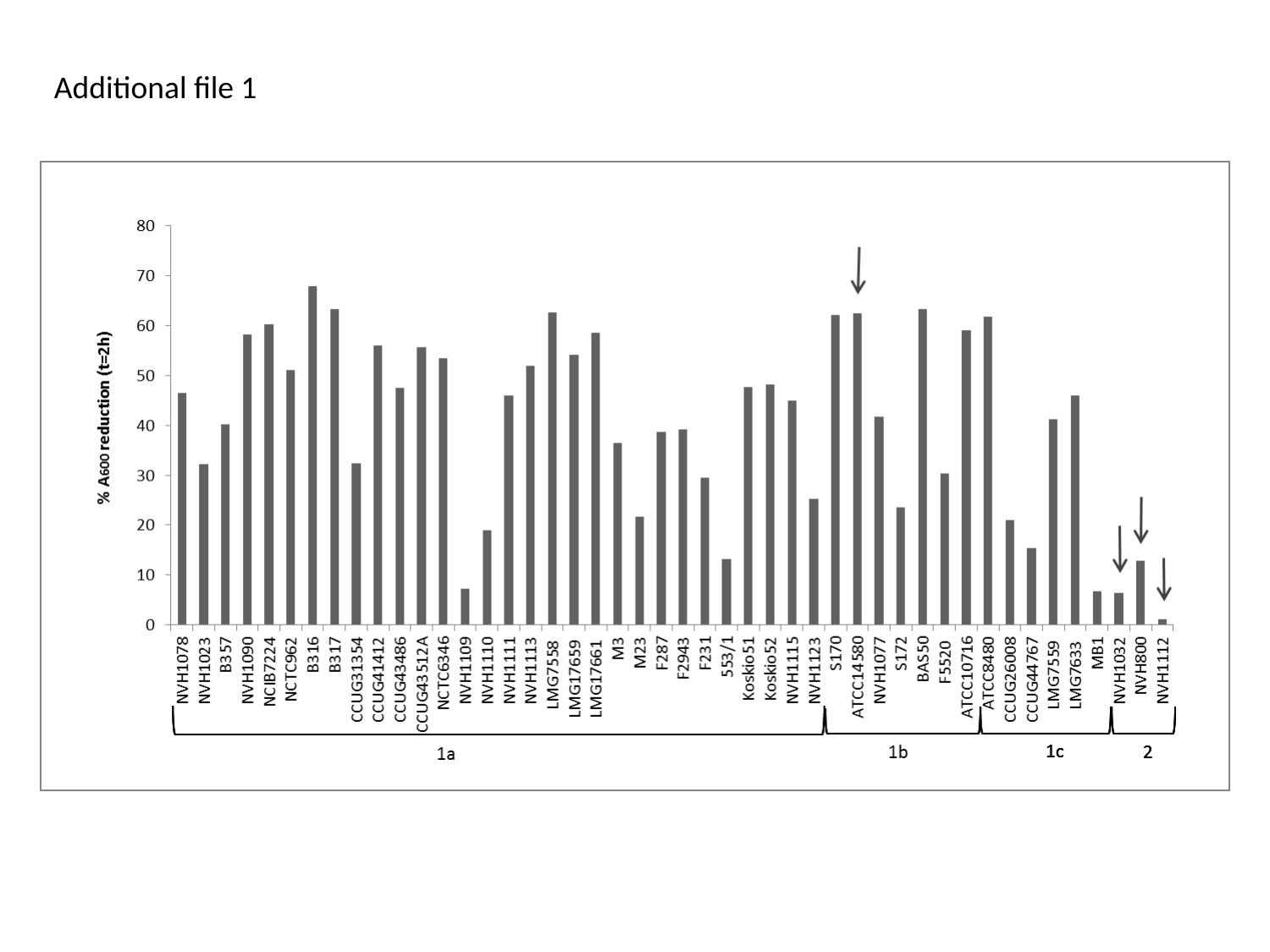

Additional file 1

Supplement: Additional file 1 — Comparison of germination efficiency in 46 B. licheniformis strains. The relative decrease in absorbance (A600) in the spore suspension was measured 2 h after the addition of germinant (100 mM L-alanine). The strains NVH1032, NVH800, ATCC14580/DSM13 and NVH1112 were selected for further analysis (indicated with arrows). [file 1471-2180-14-101-S1.pptx]

## Slide 1
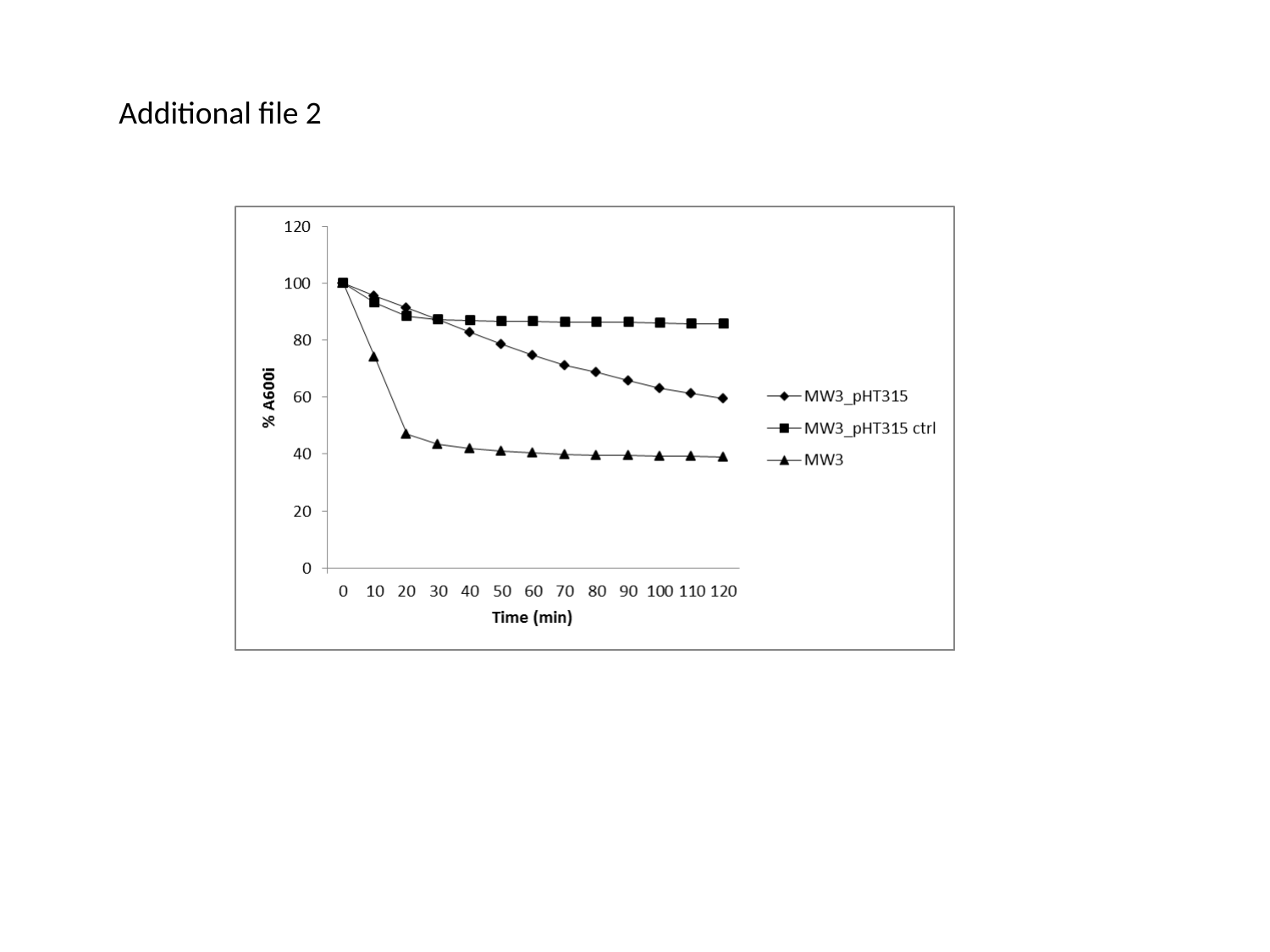

Additional file 2

Supplement: Additional file 2 — Spore germination of MW3 carrying pHT315. Germination of MW3 (▲) and MW3_pHT315 () measured as reduction in absorbance (A600) after addition of germinant (100 mM L-alanine). MW3_pHT315 ctrl (■) is not added any germinant. [file 1471-2180-14-101-S2.pptx]

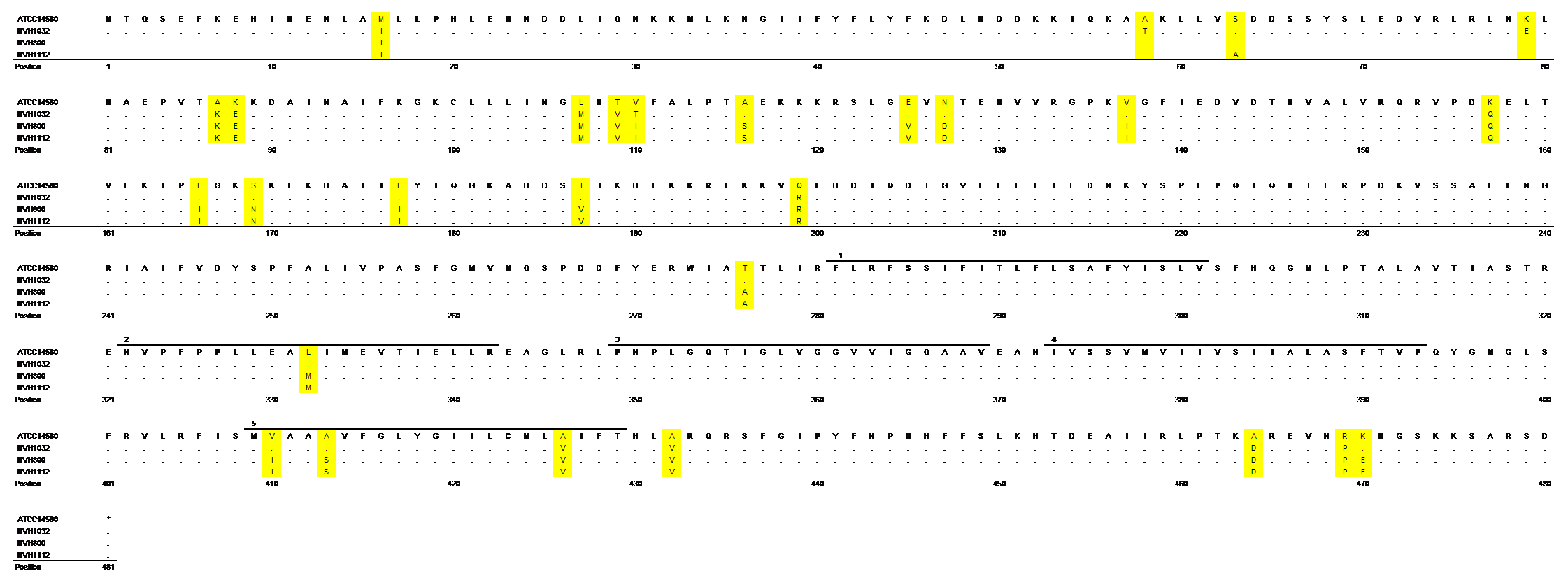

Supplement: Additional file 4 — Amino acid sequence alignment of GerAA from ATCC14580/DSM13, NVH1032, NVH800 and NVH1112. Residues with substitutions are indicated in yellow. Alignment was performed with ClustalW in MEGA5. The numbered solid lines indicate regions of predicted transmembrane domains (TOPCONS). [file 1471-2180-14-101-S4.tiff]

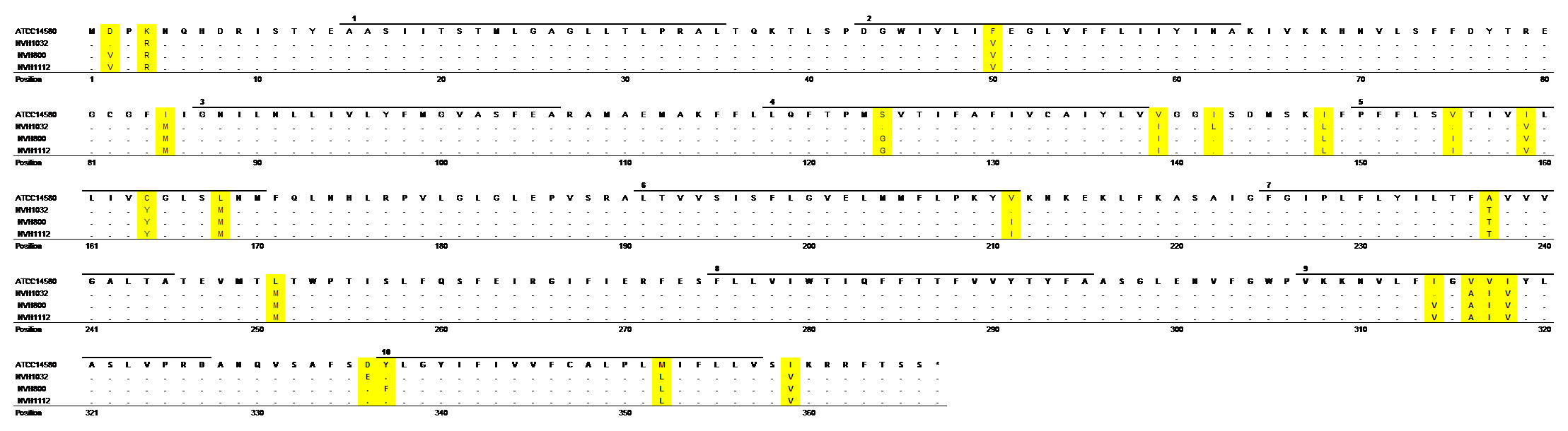

Supplement: Additional file 5 — Amino acid sequence alignment of GerAB from ATCC14580/DSM13, NVH1032, NVH800 and NVH1112. Residues with substitutions are indicated in yellow. Alignment was performed with ClustalW in MEGA5. The numbered solid lines indicate regions of predicted transmembrane domains (TOPCONS). [file 1471-2180-14-101-S5.tiff]

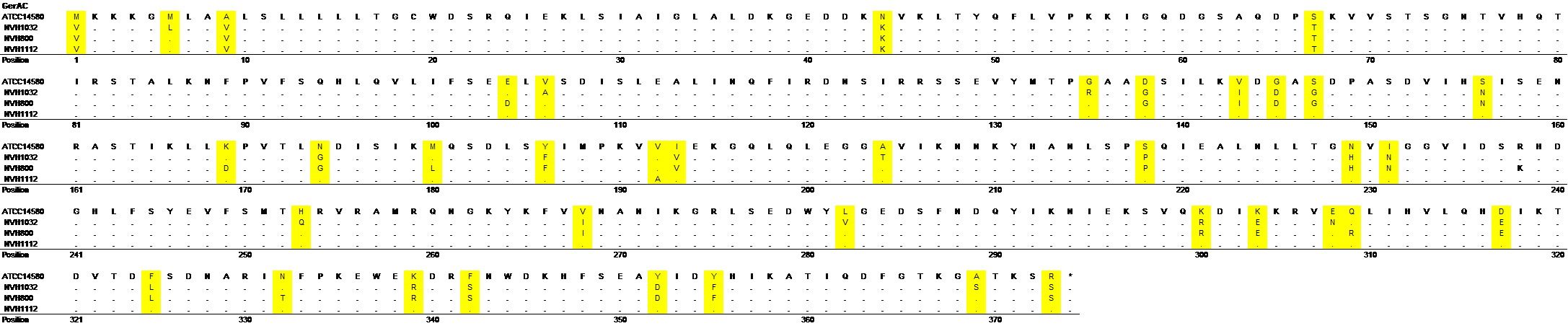

Supplement: Additional file 6 — Amino acid sequence alignment of GerAC from ATCC14580/DSM13, NVH1032, NVH800 and NVH1112. Residues with substitutions are indicated in yellow. Alignment was performed with ClustalW in MEGA5. [file 1471-2180-14-101-S6.tiff]

## Slide 1
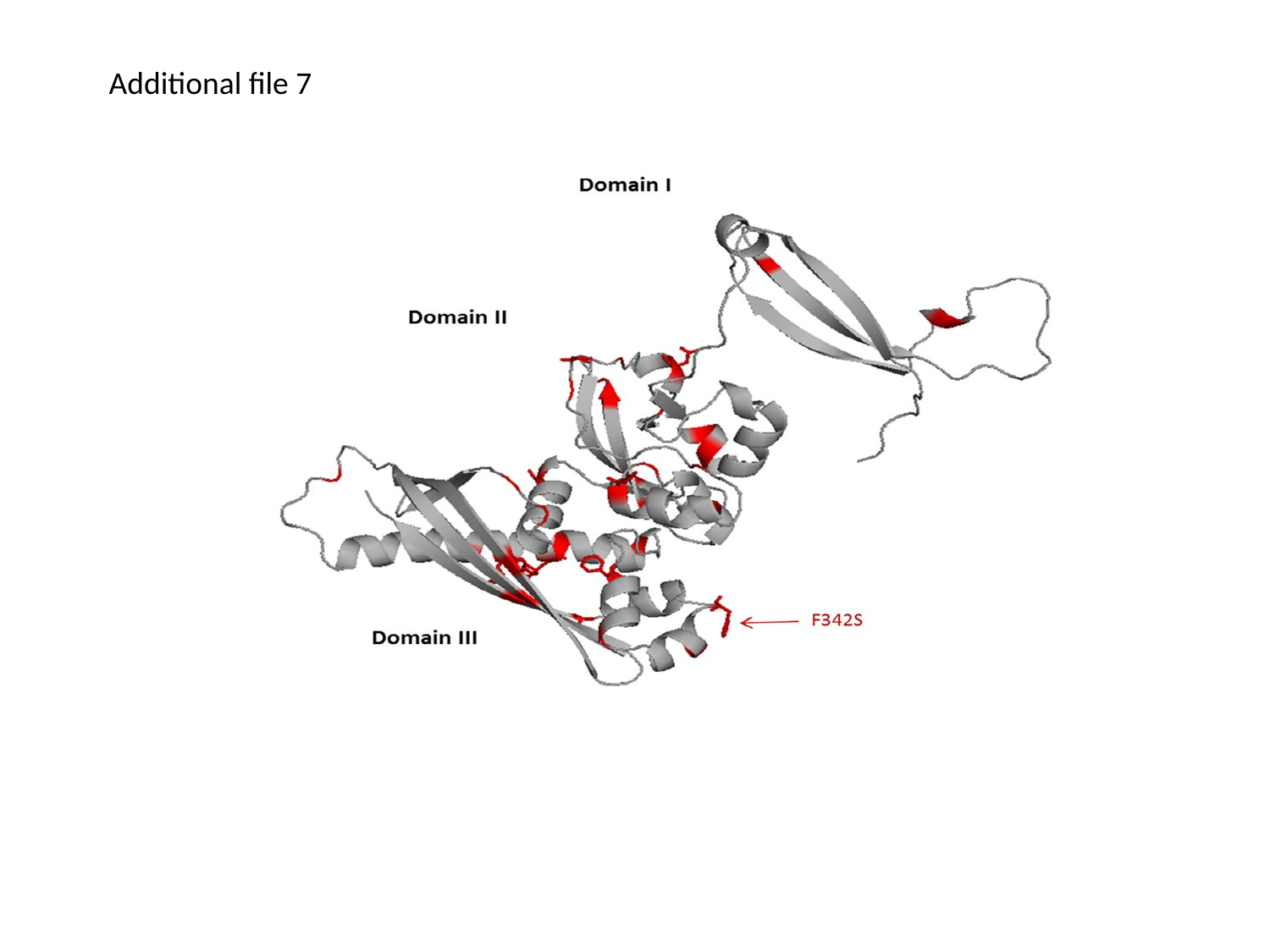

Additional file 7

Supplement: Additional file 7 — 3D-model of the GerAC protein of B. licheniformis. Substitutions that were detected in strain NVH1032, NVH800 and NVH1112 are indicated with red. Modelling was performed in PyMOL. [file 1471-2180-14-101-S7.pptx]
