# Supplementary material for: L-alanine-induced germination in Bacillus licheniformis -the impact of native gerA sequences
Source: BMC Microbiol. 2014 Apr 22;14:101. doi: 10.1186/1471-2180-14-101 (PMC4021175; doi:10.1186/1471-2180-14-101)
Supplement: Additional file 3 — Promoter sequence alignment. Alignment of the estimated σG dependent gerA promoter sequences of B. subtilis spp. subtilis str.168 and B. licheniformis ATCC14580/DSM13, NVH1112, NVH800 and NVH1032. DBTBS was used to identify promoter sequences. The B. subtilis promoter (underlined) and transcriptional start site (arrow) were experimentally defined by Feavers et al. (1990) [24]. [file 1471-2180-14-101-S3.pptx]

## Slide 1
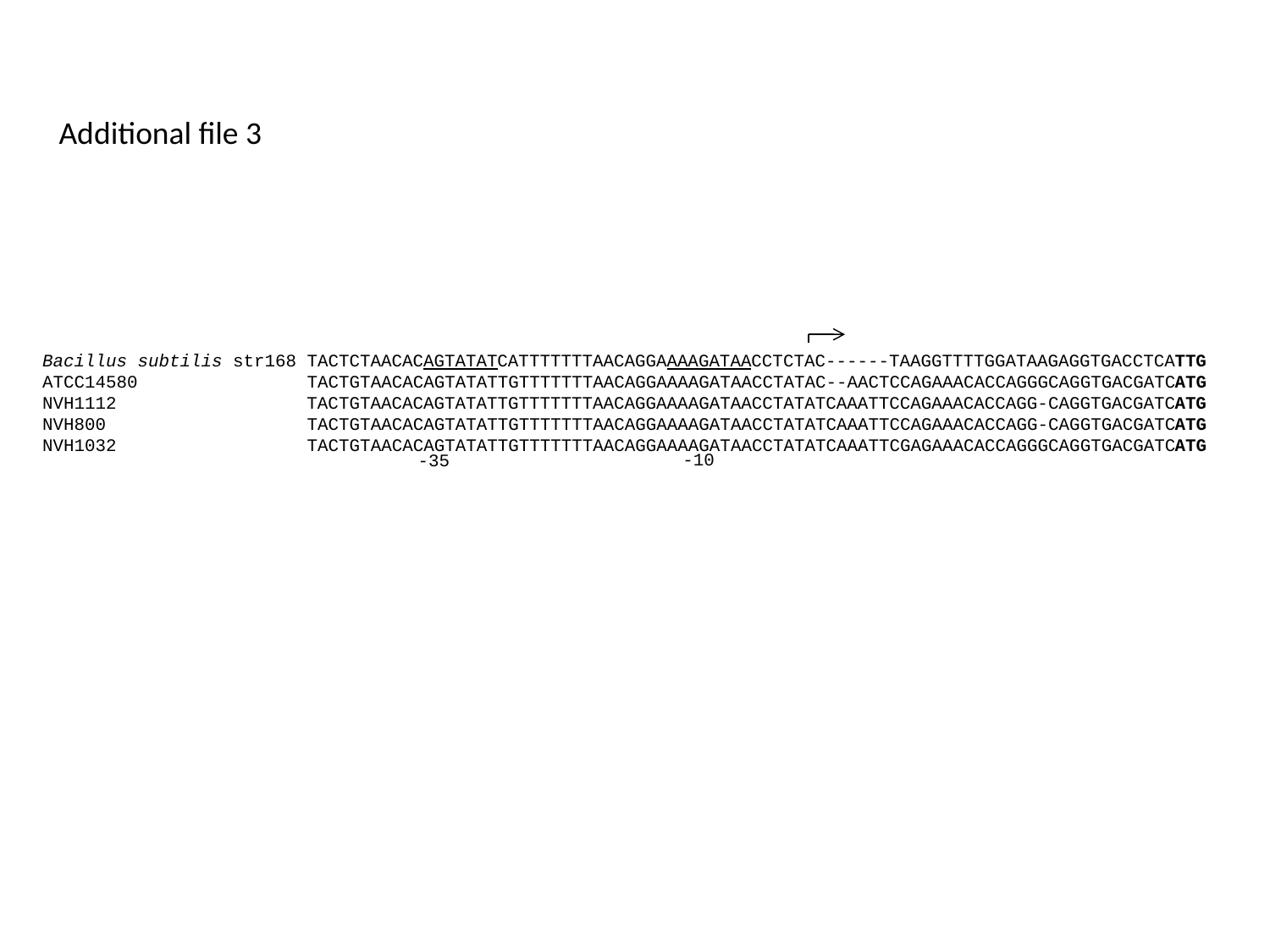

Additional file 3
Bacillus subtilis str168 TACTCTAACACAGTATATCATTTTTTTAACAGGAAAAGATAACCTCTAC------TAAGGTTTTGGATAAGAGGTGACCTCATTG
ATCC14580 TACTGTAACACAGTATATTGTTTTTTTAACAGGAAAAGATAACCTATAC--AACTCCAGAAACACCAGGGCAGGTGACGATCATG
NVH1112 TACTGTAACACAGTATATTGTTTTTTTAACAGGAAAAGATAACCTATATCAAATTCCAGAAACACCAGG-CAGGTGACGATCATG
NVH800 TACTGTAACACAGTATATTGTTTTTTTAACAGGAAAAGATAACCTATATCAAATTCCAGAAACACCAGG-CAGGTGACGATCATG
NVH1032 TACTGTAACACAGTATATTGTTTTTTTAACAGGAAAAGATAACCTATATCAAATTCGAGAAACACCAGGGCAGGTGACGATCATG
-10
-35
