# Supplementary material for: L-alanine-induced germination in Bacillus licheniformis -the impact of native gerA sequences
Source: BMC Microbiol. 2014 Apr 22;14:101. doi: 10.1186/1471-2180-14-101 (PMC4021175; doi:10.1186/1471-2180-14-101)
Supplement: Additional file 8 — Primers used in PCR amplification and DNA sequencing of gerA operons from B. licheniformis strains NVH 1112, NVH1032 and NVH800. [file 1471-2180-14-101-S8.docx]

Additional File 8

| *gerAA* operon sequencing primers | Forward primers 5’-3’ | Reverse primers 5’-3’ |
| --- | --- | --- |
| NVH1112 | APF: CTGATTTGCGCCAACTTTCT | AOR: CGCTTTGCCCTGGATATAGA |
|  | A2F: GGAACCCGTTACAGCAAAAA | A2R: CGATCCGTCCATTAAACAGC |
|  | A3F: TTCCGCAGATCCAGAATAC | A3R: GTGCTGCGGTTTATTTCCAT |
|  | A4F: CAATCCGCTAGGCCAGAC | A4R: TTTTAACGATTTTTGCATTGATGT |
|  | A1F: TCAATCTCCTCCGGTTTCC | A1R: GCGTTAATCGCGTCCTTTT |
|  | A6F: GCGGACTGAGCCTGAATATG | A6R: TCCAAGGTAGTCGCTGAAGG |
|  | A7F: GGATTTGGGATACCGCTCTT | A7R: TGCAGATGCTGCGAGAATAC |
|  | A8F: CGCTCAGGATCCGTCTAAAG | A8R: GGTGGGTCATCGAAAACACT |
|  | A9F: CAGATCGAAGCGCTGAATTT | A9R: AAAATGCCTGTCATGTGCAA |
| NVH1032 | B3F: TTCCGCAGATCCAGAATAC | B3R: ATGAAGCAAGGGCGATAATG |
|  | B1F: TCCATGAAAATCTCGCAATG | B1R: ACACGCTGCCTGACTAGAGC |
|  | B4F: CAATCCGCTAGGCCAGAC | B4R: TTTTAACGATTTTTGCATTGATGT |
|  | B9F: CAGATCGAAGCGCTGAATTT | B9R: AAAATGCCTGTCATGTGCAA |
|  | BPF: CTGATTTGCGCCAACTTTCT | BOR: CGCTTTGCCCTGGATATAGA |
|  | BSF: AAAGAAGCCTTGGAGAAGTGA | BSR: GGGTGATGAAAATCGAGCTG |
|  | B5F: TCGGATTAGCACGTATGAAGC | B5R: AAGAGCGGTATCCCAAATCC |
|  | B6F: GCGGACTGAGCCTGAATATG | B6R: TCCAAGGTAGTCGCTGAAGG |
|  | B7F: GGATTTGGGATACCGCTCTT | B7R: TGCAGATGCTGCGAGAATAC |
|  | B8F: CGCTCAGGATCCGTCTAAAG | B8R: GGTGGGTCATCGAAAACACT |
| NVH800 | C1F: TCAATCTCCTCCGGTTTCC | C1R: GGCAGCGCAAAAATAACATT |
|  | C2F: GGAACCCGTTACAGCAAAAA | C2R: CGATCCGTCCATTAAACAGC |
|  | C2F: GGAACCCGTTACAGCAAAAA | C2R: CGATCCGTCCATTAAACAGC |
|  | C3F: TTCCGCAGATCCAGAATAC | C3R: ATGAAGCAAGGGCGATAATG |
|  | C4F: CAATCCGCTAGGCCAGAC | C4R: TTTTAACGATTTTTGCATTGATGT |
|  | C5F: TCGGATTAGCACGTATGAAGC | C5R: AAGAGCGGTATCCCAAATCC |
|  | C6F: GCGGACTGAGCCTGAATATG | C6R: TCCAAGGTAGTCGCTGAAGG |
|  | C7F: GGATTTGGGATACCGCTCTT | C7R: TGCAGATGCTGCGAGAATAC |
|  | C8F: CGCTCAGGATCCGTCTAAAG | C8R: GGTGGGTCATCGAAAACACT |
|  | C9F: CAGATCGAAGCGCTGAATTT | C9R: AAAATGCCTGTCATGTGCAA |
|  | C10F: GAAGGCTGTCAGAGGACTGG | C10R: AACGGATCATAGGCGACATC |
